# Crystal engineering via mechanochemistry: Cocrystals, salts, and polymorphs

**DOI:** 10.1063/4.0000989

**Published:** 2025-10-27

**Authors:** Delbert S Botes, Kristin Hutchins

**Affiliations:** University of Missouri, Columbia, Missouri, USA

## Abstract

Crystal engineering provides exciting opportunities in solid-state materials development, especially when considering small molecule active pharmaceutical ingredients (APIs). Cocrystallization of an API with an applicable coformer can modify many of the API’s physicochemical characteristics such as solubility, stability, compatibility, permeability and bioavailability. This is particularly important when considering that the majority of APIs are administered orally with many possessing poor properties. Supramolecular synthons, which exploit non-covalent interactions like hydrogen bonding, are used to cocrystallize molecules together and these resulting multicomponent crystals (cocrystals and salts) have proven their tremendous utility. Polymorphism, the ability of an API to crystallize in different crystal forms each with its own unique properties, also has important implications in pharmaceutical development. Being able to control the polymorphism and deliver an optimal API is essential as vividly illustrated by the HIV drug ritonavir. Typically, generating these different crystal forms, multicomponent crystals and polymorphs, involve solution-based methods. However, mechanochemistry has re-emerged as a green and robust method for their formation and has proven to be a useful part of the toolkit of crystal engineers. Here we report on the assembly of multicomponent crystals of APIs not extensively studied as well as efforts towards controlling polymorphism, all via mechanochemistry.

